# A Simple yet Efficient Water-Saving Condenser

**DOI:** 10.1021/acsomega.5c02494

**Published:** 2025-08-02

**Authors:** Stéphane Rosset, Clément Mazet

**Affiliations:** Department of Organic Chemistry, 27212 University of Geneva, 30 Quai Ernest Ansermet, 1211 Geneva, Switzerland

## Abstract

We describe the design of an efficient waterless condenser for use in chemical synthesis, which exists in two different sizes. This laboratory equipment displays performances comparable to those of conventional water-jacketed glass condensers across a broad range of organic solvents. It is most effective when fitted with a Teflon cap containing a safety valve. The condenser with the smallest cross-section is particularly suitable for reactions using water as a solvent, despite its lower vapor density than air.

## Graphical abstract

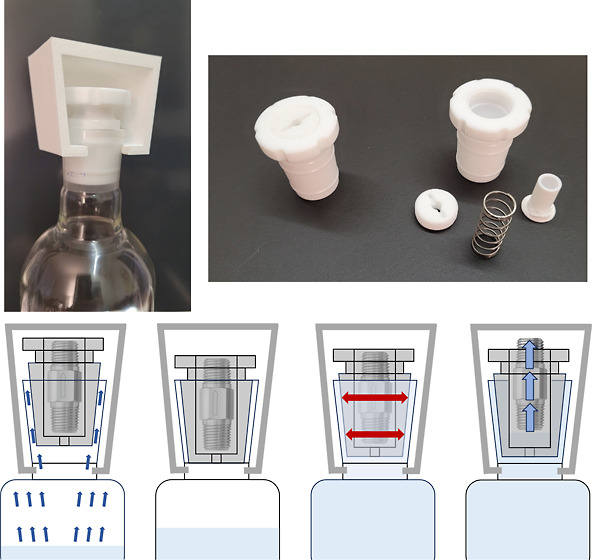

## Introduction

Water is a precious resource that is essential to life. Water is also indispensable in most research laboratories. Although there is a growing awareness of water waste and creative approaches are regularly proposed to researchers to optimize its daily consumption, conservatism and a limited number of practical solutions are typical obstacles to changing (bad) practices.^1–10^

In synthetic chemistry research laboratories, water is typically used as a solvent, for extractions and removal of byproducts, to precipitate solids, or as a liquid for cooling baths. A large amount of water is also consumed for cleaning reaction vessels and glassware or to ensure the functioning of numerous instruments. A major source of water consumption is linked to the cooling of reactions that are run under reflux, distillations, and rotary evaporations. To replace the conventional water-jacketed glass condensers (type **A**, Figure 1), a number of alternatives have emerged over the last decades (among which are types **B**–**C**, Figure 1).^11,12^ Water-jacketed glass condensers typically operate with continuous water flow, often for extended time periods. Besides the obvious negative ecological impact, this is a frequent source of flooding. Despite the demonstrated efficiency of the recent water-free condensers for a majority of common organic solvents, their use has not become widespread.^13,14^ This is presumably due to their prohibitive costs and their moderate performances for low-boiling solvents (i.e., rapid saturation).

**Figure 1:**
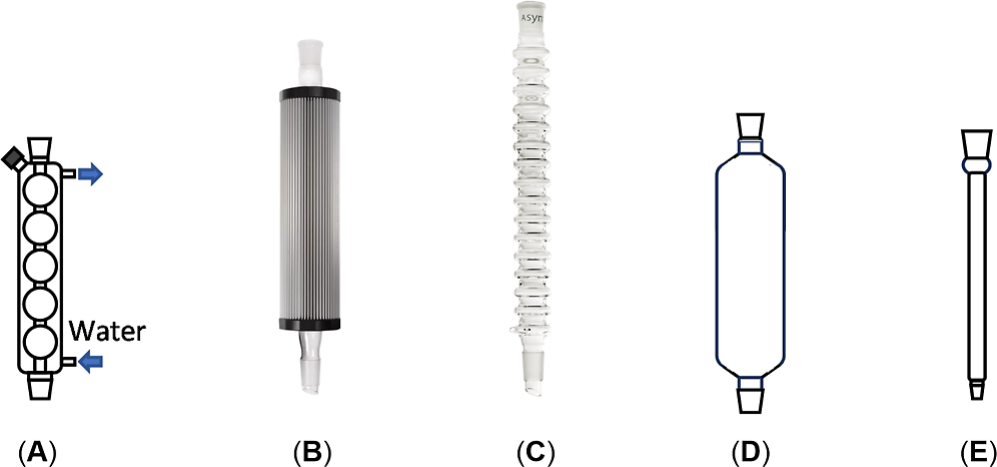
Water condensers: (A) water-jacketed Allihn condenser. (B) Findenser. (C) Condensyn. (D) RoMa prototype (large). (**E**) RoMa prototype (small).

We report herein the development of a simple and practical water-free glass condenser. This user-friendly laboratory equipment exists in two different sizes depending on the volume of solvent employed (types **D**–**E**, Figure 1). For optimal efficiency, it can be fitted with a Teflon stopper containing a safety valve. This system is compatible with an array of standard organic solvents and compares favorably with traditional water-jacketed glass condensers. We also show that water can be used as a solvent despite its lower vapor density than air.

## Material and Methods

Solvents (ACS grade) were purchased from commercial suppliers and used as received. All experiments were run using a Heidolph MR Hei-Tec digital hot plate stirrer. Volumetric measurements were made at room temperature (ca. 20–23 °C) using graduated glass cylinders, 500 mL (±2 mL), 250 mL (±1 mL), 50 and 25 mL (±0.5 mL), and 10 mL (±0.2 mL). In order to limit random losses, all tests were carried out using polytetrafluoroethylene (PTFE) sleeves on the condensers ground glass cone. As the energy delivered to the solvent is highly dependent on the interface between the flask and its environment, this must be perfectly controlled to maximize the reproducibility. Tests performed on the 50, 100, and 250 mL round-bottom flasks were carried out using heating blocks and inserts corresponding to the volume of the flask, in which silicone oil was added to limit the effect of the geometry of the different flask. The tests carried out on 500 mL of solvents were carried out using always the same 1 L flask in an oil-free heating block. Two types of DrySyn heating blocks were used (see Supporting Information). The tests on the 10 mL flask were carried out in oil baths. All tests were carried out with the sash of the fumehood closed.

## Results and Discussion

Traditionally, air-cooled condensers are designed so that the solvent vapors to be contained are in close contact with a large glass surface, a surface that must then efficiently dissipate the heat transferred. The need to funnel all vapors close to the glass, while at the same time providing a large heat exchange surface, generally requires the use of a fairly sophisticated glass shape. Based on the observation that all organic solvent vapors have a higher density than air, we hypothesized that cross-sectional constriction is not fundamental to the design of an efficient condenser. The hypothesis was tested by simplifying the condenser geometry to the extreme, using a simple ground glass tube with a diameter of 7 cm and a length of 20 cm (type **D**, Figures 1 and 2A). These dimensions ensured that the internal exchange surface (400 cm^2^) was close to that of a reference system (Findenser: type **B**, Figure 1), while offering an internal volume five times greater (700 cm^3^ vs 130 cm^3^) (see Supporting Information for detailed specifications).

**Figure 2:**
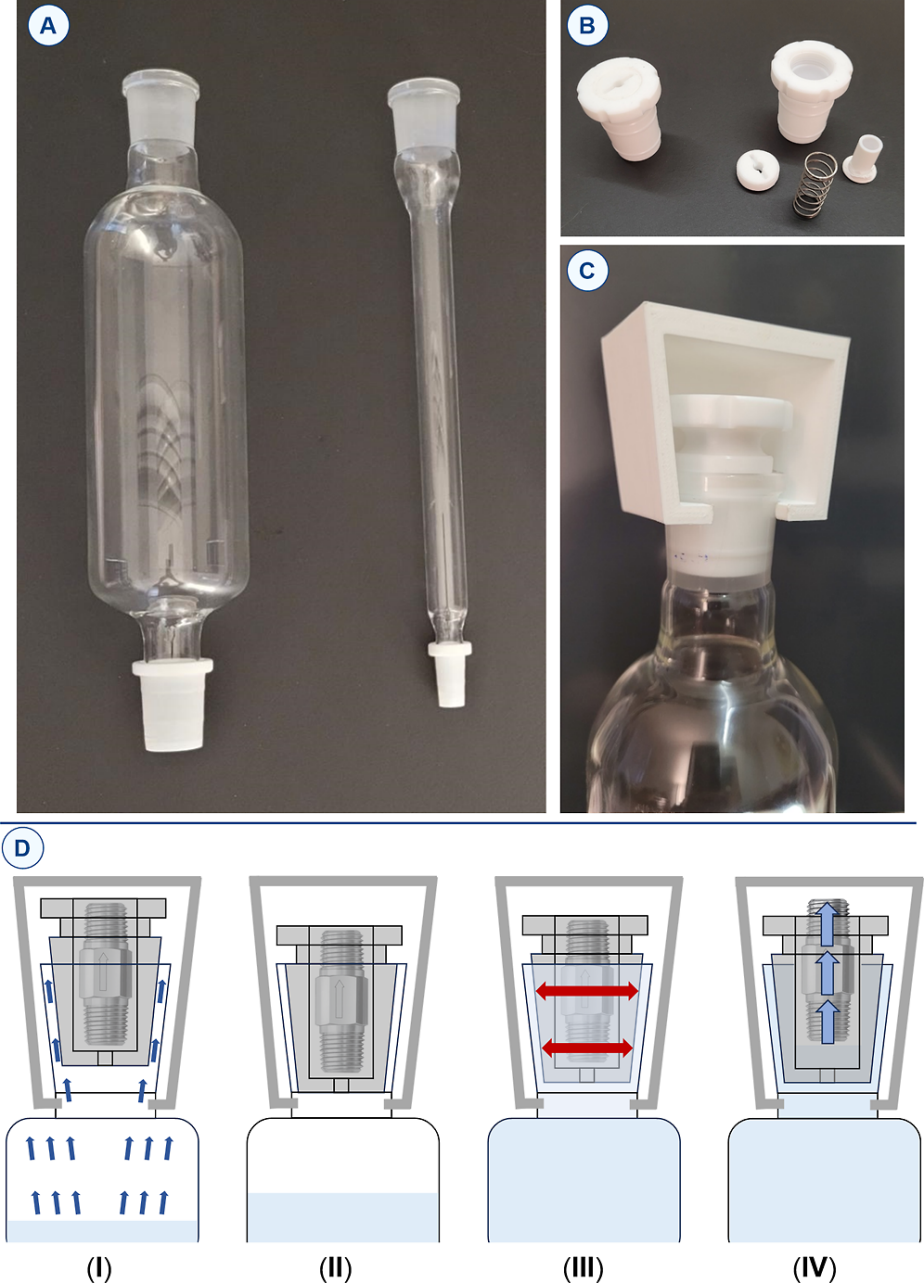
(A) RoMa prototypes with PTFE sleeves. (B) Assembled and disassembled PTFE stopper equipped with a safety valve. (C) Large RoMa prototype equipped with PTFE stopper and stop device. (D) Operating regimes of the stopper: (I) gas purge (blue arrows). (II) Equilibrium. (III) Exchange surface saturation and thermal expansion of the stopper (red arrows). (IV) Valve opening (*P* > 0.15 bar above *P*_atm_).

We began our investigations by comparing the performance of our device (**D**) to that of the commercially available Findenser (**B**) and of a regular water-jacketed Allihn condenser (**A**), using four organic solvents commonly employed in synthetic laboratories with distinct boiling points: dichloromethane (40 °C), acetone (56 °C), ethyl acetate (77 °C), and toluene (111 °C). Half-filled 1 L round-bottom flasks were heated significantly above the boiling point of each solvent (10–25 °C), and the volume loss was measured after 18 h (Table 1). In all cases, the best performances were obtained with the conventional water-jacketed condenser (**A**) (entries 1, 4, 7, and 10). While total solvent loss was observed with condensers **B** and **D** using dichloromethane (entries 2–3), saturation was only observed with **B**, using acetone and ethyl acetate (entries 5, 8). The simple glass condenser (**D**) led to much reduced losses of 15% and 2%, respectively (entries 6, 9). As expected for a high-boiling point solvent, similar results were obtained between all three systems with refluxing toluene, with only minimal evaporation noticed after 18 h (entries 10–12).

**Table 1:** Solvent Loss Using 500 mL after 18 h at Reflux^a^

entry	solvent	bp/T (°C)^b^	condenser	loss (mL)	% loss
1	CH_2_Cl_2_	40/50	**A**	5	1
2	CH_2_Cl_2_	40/50	**B**	500	100
3	CH_2_Cl_2_	40/50	**D**	500	100
4	Me_2_CO	56/71	**A**	5	1
5	Me_2_CO	56/71	**B**	500	100
6	Me_2_CO	56/71	**D**	75	15
7	EtOAc	77/97	**A**	5	1
8	EtOAc	77/97	**B**	500	100
9	EtOAc	77/97	**D**	10	2
10	toluene	111/136	**A**	5	1
11	toluene	111/136	**B**	5	1
12	toluene	111/136	**D**	5	1
13	CH_2_Cl_2_	40/50	**D.v** ^c^	10	2
14	Me_2_CO	56/71	**D.v** ^c^	5	1
15	EtOAc	77/97	**D.v** ^c^	5	1
16	toluene	111/136	**D.v** ^c^	5	1

a Reactions performed in 1 L round-bottom flasks filled with 500 mL of the indicated solvent.

b bp: boiling point. *T*: heating temperature.

c Condenser equipped with the PTFE stopper.

Following these preliminary results, we set out to optimize the performance of condenser **D**. Because organic solvent vapors are heavier than the gas in the glass condenser (whether air, nitrogen, or argon), they first condense at the bottom of the system before rising to the upper outlet, thus expelling the gas initially present in the condenser and eventually solvent vapors. In order to reduce the loss of residual solvent vapors without increasing the internal pressure in the glass apparatus, we designed a PTFE stopper containing an internal safety valve (Figure 2).^15^ A commercially available PTFE stopper (COWIE, item #010.229),^16^ to be placed on the top outlet of the condenser, was modified by inserting a safety valve in its center, reminiscent of a pressure cooker system. In the case of moderate heating, such as those applied during our tests, an equilibrium is established between the flow of vapors generated and that of condensation, involving only a fraction of the available glass cooling surface (Figure 2D­(I,II)). We observed that when the vapors reach the top of the apparatus, the PTFE stopper expands, inducing hermetic closure of the device and preventing solvent loss (Figure 2D­(III)). If excessive heating is inadvertently triggered, the overpressure generated is relieved by the built-in safety valve, which opens when the internal pressure exceeds the atmospheric pressure by 0.15 bar or more (Figure 2D­(IV)). To further reinforce safety, would the PTFE stopper be expelled during heating, a stop device that clips around the top neck of the condenser was also designed (Figure 2B,C). Importantly, such a situation has never occurred in the many tests we have carried out, regardless of the nature of the solvent used.

We observed that when the large condenser (**D**) was used together with the PTFE stopper (noted **D.v**), performances similar to those measured with a conventional water-jacketed condenser (**A**) were obtained for all four solvents (Table 1, entries 13–16). We next performed a comparative study using the same set of solvents with volumes ranging from 125 to 5 mL in half-filled round-bottom flasks (Table 2). With solvent volumes of 125 or 50 mL, the results obtained with the newly developed glass condenser mounted with the PTFE stopper paralleled favorably with the conventional water-jacketed condenser (**A**) (entries 1 vs 4 and 5 vs 8). By contrast, condensers **B** and **C** reached saturation for dichloromethane, leading to complete evaporation after 18 h at reflux. Nonetheless, they maintained appreciable performances with ethyl acetate and toluene (entries 2–3 and 6–7).

**Table 2:** Solvent Loss Measured for Volumes of 125 mL, 50 mL, and 25 mL after 18 h at Reflux^a^

			solvent loss (mL)			
Entry	condenser	volume of solvent (mL)	CH_2_Cl_2_ (40/50)^b^	Me_2_CO (56/71)^b^	EtOAc (77/97)^b^	toluene (111/136)^b^
1	**A**	125	4	1	1	1
2	**B**	125	125	5	1.5	1
3	**C**	125	125	5	1.5	1
4	**D.v** ^c^	125	4	1	1	1
5	**A**	50	1.5	1.0	0.5	0.5
6	**B**	50	50	3.5	1.5	0.5
7	**C**	50	50	3.5	1.5	0.5
8	**D.v** ^c^	50	3.0	1.0	1.0	0.5
9	**A**	5	1.0	0.6	0.4	0.2
10	**B**	5	3.4	1.4	0.6	0.2
11	**C**	5	3.0	1.4	0.4	0.4
12	**E.v** ^c^	5	0.6	0.4	0.2	0.2

a Reactions performed in half-filled round-bottom flasks with the indicated solvent.

b First value in parentheses: boiling point. Second value in parentheses: heating temperature.

c Condenser equipped with the PTFE stopper.

The smallest prototype (noted **E** or **E.v**) was next employed for very small volumes of solvent (5 mL). Its length is identical to that of **D** (20 cm) but its diameter is only 2 cm, still providing an internal volume of 50 cm^3^. The upper outlet permits a connection to the PTFE stopper containing the internal safety valve. While the results are essentially similar between the three condensers tested (**B**, **C**, and **E.v**) for ethyl acetate and toluene, the newly designed condenser equipped with the PTFE stopper (**E.v**) clearly outperforms **B** and **C** for acetone and dichloromethane, the lightest solvents, with only minimal evaporation over 18 h (Table 2, entries 10–12).

From a conceptual point of view, the condensers were designed on the assumption that organic solvent vapors have a higher density than air. Consequently, we assumed that the use of water as a solvent could be particularly problematic, as its vapor density is lower than that of air. To address this potential concern, we evaluated the efficiency of the condensers using water and 1,4-dioxane as solvents, both of which have a similar boiling point (100 and 101 °C, respectively) but a different vapor density (0.8 and 3.0, respectively). These tests were run under particularly demanding conditions with a heating temperature of 125 °C for 18 h (Table 3).

**Table 3:** Solvent Loss after 18 h at 125 °C^a^

			solvent loss (mL)	
entry	condenser	*V*_solvent_ (mL)	water (100/125)^c^	1,4-dioxane (101/125)^c^
1	**D**	500	90	5
2	**D.v** ^b^	500	2	2
3	**D**	25	24	2.5
4	**D.v** ^b^	25	1.0	1.0
5	**E**	25	0.5	0.5
6	**E.v** ^b^	25	0.5	0.5

a Reactions performed in half-filled round-bottom flasks with the indicated solvent.

b Condenser equipped with the PTFE stopper.

c First value in parentheses: boiling point. Second value in parentheses: heating temperature.

Consistent with our previous results, we found that only a minimal amount of 1,4-dioxane was lost, regardless of the initial volume and the use or not of the PTFE stopper equipped with the safety valve. In contrast, for a reaction using 500 mL of water, we noticed a substantial loss of solvent after 18 h (ca. 20%, entry 1). We could remediate this problem by using the condenser equipped with the PTFE stopper (**D.v**, entry 2). For reactions carried out with a volume of only 25 mL, complete evaporation was observed when using condenser **D** without the stopper (entry 3). The use of the PTFE stopper (**D.v**) led to a solvent loss of about 4% (entry 4). We noticed that unlike organic solvent vapors, which rise from the bottom to the top of the condenser with a well-defined horizontal front, water vapors rise more diffusely. Consequently, the smaller cross-section of condenser **E** compared with **D** (Ø = 2 cm vs Ø = 7 cm) should considerably reduce water vapor loss by favoring their contiguity with the glass wall. The results presented in entries 5 and 6 of Table 3 validate this hypothesis, since only 2% of the water evaporated when using **E** with or without the PTFE stopper.

## Conclusion

In summary, we developed a simple and practical water-free glass condenser to be used for standard reactions that are run under reflux in synthetic chemistry research laboratories. This equipment was designed in two different sizes to adapt to the volume of solvent used. Under the heating conditions employed, this system compares favorably with traditional water-jacketed glass condensers for a broad range of organic solvents with high and low boiling points. Its efficacy is optimal when fitted with a Teflon stopper with a safety valve. Finally, we established that the condenser with the smallest cross-section is particularly well-suited for reactions using water as a solvent notwithstanding its lower vapor density relative to air.

## Supplementary Material



## Notes

Additional experimental details, materials, and methods, including photographs of experimental setup (PDF)
