# Supplementary material for: A Simple yet Efficient Water-Saving Condenser
Source: ACS Omega. 2025 Aug 2;10(31):34438–41. doi: 10.1021/acsomega.5c02494 (PMC12355254; doi:10.1021/acsomega.5c02494)
Supplement: Supplementary file 1 [file ao5c02494_si_001.pdf]

A Simple yet Efficient Water-Saving Condenser

Stéphane Rosset[†] and Clément Mazet[†]*

[†] Department of Organic Chemistry, University of Geneva, 30 quai Ernest Ansermet, 1211 Geneva, Switzerland.

clement.mazet@unige.ch

Table of Contents

1. General information 3

2. The five different types of condensers used in this study 3

3. Heating blocks used in this study 6

4. Additional data 7

Table S1. Solvent loss measured after 18 h at reflux (additional data) 7

5. References 7

1. General information

Solvents (ACS grade) were purchased from commercial suppliers and used as received. All experiments were run using Heidolph MR Hei-Tec digital hot plate stirrer. Volumetric measurements were made at room temperature using graduated glass cylinders, 500 mL (± 2 mL), 250 mL (± 1 mL), 50 mL and 25 mL (± 0.5 mL), 10 mL (± 0.2 mL). In order to limit random losses all tests were carried out using PTFE sleeves on the condensers ground glass cone. As the energy delivered to the solvent is highly dependent on the interface between the flask and its environment, this must be perfectly controlled to maximize reproducibility. Tests performed on the 50 mL, 100 mL and 250 mL round bottom flasks were carried out using heating blocks and inserts corresponding to the volume of the flask, in which silicone oil was added to limit the effect of the geometry of the different flask. The tests carried out on 500 mL of solvents were carried out using always the same 1 L flask in an oil-free heating block. Two types of DrySyn heating blocks were used (as stated in item 3 of this SI). The tests on the 10 mL flask were carried out in oil baths. All tests were carried out with the sash of the fumehood closed.

2. The five different types of condensers used in this study

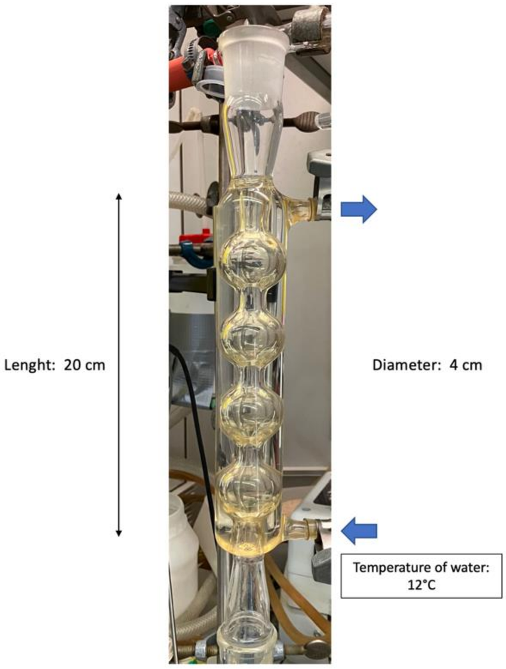

Figure S1. Water-jacketed Allihn condenser (**A**)

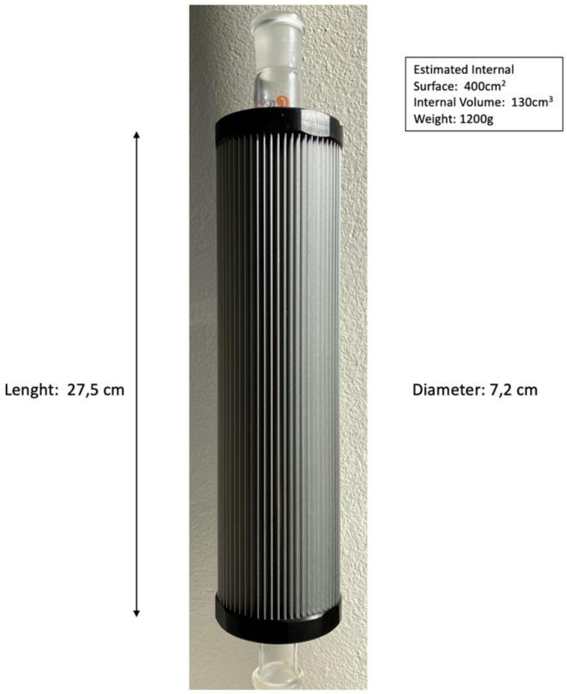

Figure S2. Air condenser Findenser (**B**)[1]

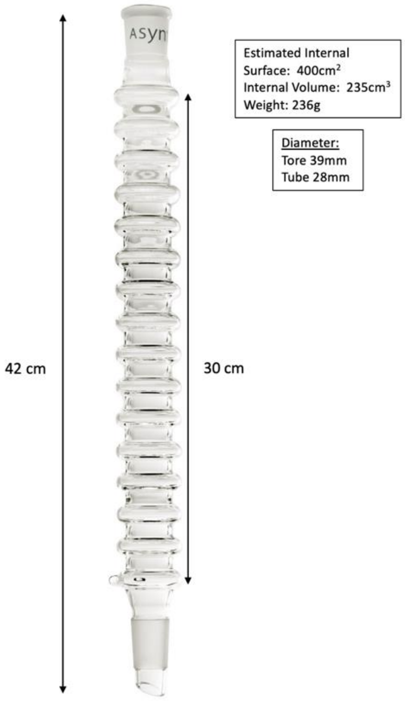

Figure S3. Air condenser Condensyn (**C**)[2]

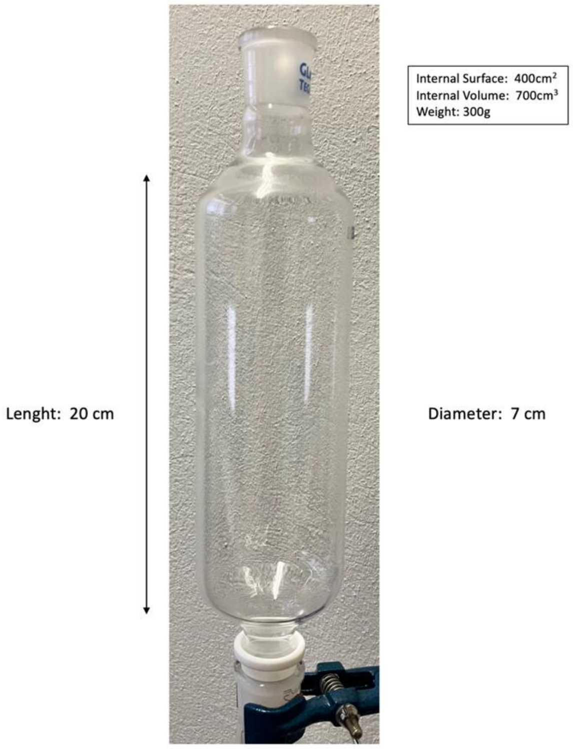

Figure S4. Air condenser RoMa large (**D**)

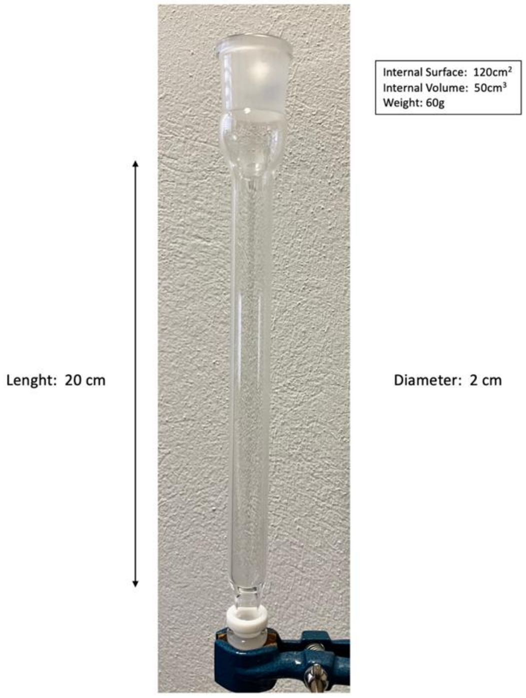

Figure S5. Air condenser RoMa small (**E**)

3. Heating blocks used in this study

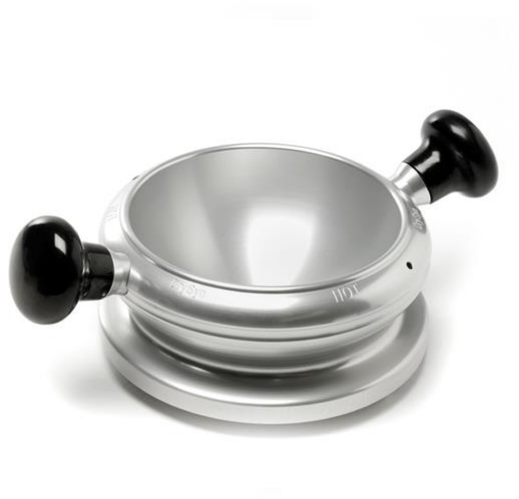

Figure S6. DRYSYN used to perform the reactions reported in Table 1 of the manuscript.

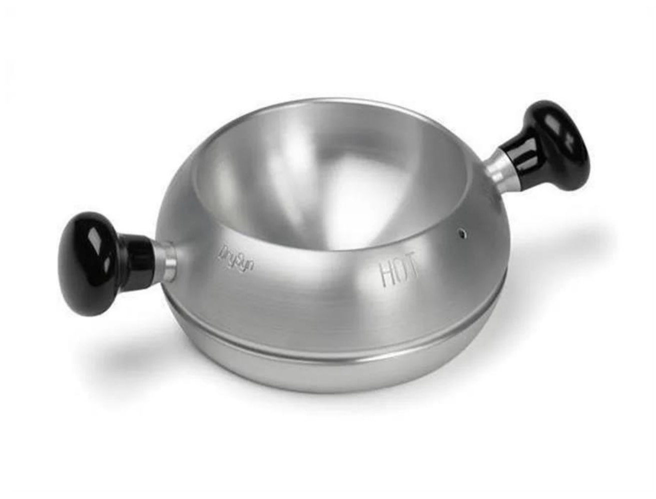

Figure S7. DRYSYN used to perform the reactions reported in Tables 2-4 of the manuscript and Tables S1.

4. Additional data

Table S1. Solvent loss measured after 18 h at reflux (additional data)[a]

entry	condenser	volume of solvent (mL)	solvent loss (mL)		
			CH_2Cl_2 (40/50)[b]	Me_2CO (56/71)[b]	EtOAc (77/97)[b]
1	**B**	5	3.4	1.4	0.6
2	**C**	5	3.0	1.4	0.4
3	**E.v**	5	0.6	0.4	0.2
4	**E**	5	3.0		
5	**B**	13	3.5	2.5	
6	**C**	13	3.0		
7	**E.v**	13	1.0	0.5	
8	**E**	13	4.5	1.5	
9	**B**	25	25	2.5	
10	**C**	25	5.5	2.5	
11	**D.v**	25	2.5	1.0	
12	**D**	25	7.5		
13	**B**	50	50	3.5	1.5
14	**C**	50	50	3.5	1.5
15	**E.v**	50	50	50	1.0
16	**D.v**	50	3.0	1.0	1.0
17	**D**	50	50	11	4.5

[a] Reactions performed in half-filled round bottom flasks with the indicated solvent. [b] First value: boiling point. Second value: heating temperature.

5. References

(1) *Findenser Waterless Air Condenser; Available from:* https://www.radleys.com/range/findenser-super-air-condenser/ (accessed March 7, 2025).

(2) *Condensyn Waterless Air Condenser; Available from:* https://www.asynt.com/product/asynt-condensyn-air-condenser/ (accessed March 7, 2025).
